# Effects of Traditional Chinese Medicine on Chemotherapy-Induced Myelosuppression and Febrile Neutropenia in Breast Cancer Patients

**DOI:** 10.1155/2015/736197

**Published:** 2015-08-12

**Authors:** Huan Tian, Wei Qin, Wenjing Wu, Pi Guo, Yong Lu, Pengxi Liu, Qiang Liu, Fengxi Su

**Affiliations:** ^1^Department of Breast Surgery, Sun Yat-sen Memorial Hospital, Sun Yat-sen University, 107 Yanjiangxi Road, Guangzhou 510120, China; ^2^Department of Breast Surgery, Guangdong Hospital of Traditional Chinese Medicine, Traditional Chinese Medicine University of Guangzhou, 111 Dade Road, Guangzhou 510120, China; ^3^Department of Hepatic Surgery, The Third Affiliated Hospital, Sun Yat-sen University, 600 Tianhe Road, Guangzhou 510630, China; ^4^Department of Medical Statistics and Epidemiology, School of Public Health, Sun Yat-sen University, 74 Zhongshaner Road, Guangzhou 510080, China

## Abstract

*Title*. Chemotherapy-induced myelosuppression lowers the quality of life in breast cancer patients and causes many complications. Traditional Chinese Medicine (TCM) is a widely used complementary and alternative medicine therapies. *Objective*. To study whether TCM can reduce the incidence of chemotherapy-induced leukopenia, neutropenia, and febrile neutropenia (FN) in breast cancer patients. *Methods*. The data were analyzed retrospectively between patients who received TCM treatment (group 1, *n* = 453) and patients who did not receive TCM treatment (group 2, *n* = 359). Significant risk factors associated with the occurrence of chemotherapy-induced leukopenia, neutropenia, and FN were identified using multivariate analysis. Propensity score-matched patients were analyzed to adjust for any baseline differences. *Results*. Group 1 patients had a significantly lower rate of chemotherapy-induced severe leukopenia, neutropenia, and FN, compared with group 2 (43% versus 71%, *P* < 0.0001, 72% versus 78%, *P* = 0.005, 6% versus 24%, *P* < 0.0001, resp.). Multivariate analysis revealed that chemotherapy regimens containing anthracyclines combined with paclitaxel or docetaxel were the most significant predictor. Subgroup analysis indicated that TCM treatment showed benefit in relieving chemotherapy-induced leukopenia and FN in most chemotherapy regimens. *Conclusions*. TCM treatment could lower the risk of severe chemotherapy-induced leukopenia, neutropenia, and FN in breast cancer patients.

## 1. Introduction

Chemotherapy is one of the major categories of pharmacotherapy for breast cancer. Chemotherapy-induced leukopenia, neutropenia, and FN are the most common and dose-limiting toxicity of cytotoxic anticancer agents and often increase the susceptibility to the development of infections in breast cancer patients [1, 2]. Moreover, severe leukopenia, neutropenia, and FN often result in treatment delay or dose reduction and discontinuation, which ultimately may compromise the long-term clinical outcome and lower the disease-free and overall survival rates [3–5].

TCM has a long history of application to disease treatment in China and has been used in numerous patients [6]. Some herbal components have also demonstrated antitumor activity by improving immune function [7]. Previous studies have demonstrated that* Oldenlandia diffusa* or* Scutellaria barbata* have antiproliferative effects on breast cancer cells in vitro and promising clinical effects in patients [8, 9]. However, there is no report regarding the benefit of TCM in alleviating hematotoxicity. The aim of our study is to determine whether TCM can reduce the incidence of chemotherapy-induced leukopenia, neutropenia, and FN in breast cancer patients.

## 2. Materials and Methods

This study was approved by the Ethics Committees of Sun Yat-sen Memorial Hospital and Guangdong Hospital of Traditional Chinese Medicine, China. It was conducted in accordance with the Declaration of Helsinki.

### 2.1. Patient Population

This study was carried out on medical records of patients diagnosed with breast cancer who had been included, between January 2011 and April 2014, in prospective databases at Sun Yat-sen Memorial Hospital and Guangdong Hospital of Traditional Chinese Medicine, China. During the study period, 507 breast cancer patients from Guangdong Hospital of Traditional Chinese Medicine, and 521 from Sun Yat-sen Memorial Hospital were enrolled in the databases. Complete data were available from only 453 breast cancer patients in Guangdong Hospital of Traditional Chinese Medicine and 359 breast cancer patients in Sun Yat-sen Memorial Hospital. Breast cancer diagnosis was confirmed by needle biopsy or surgery.

### 2.2. Entry and Exclusion Criteria

Only patients who fulfill the following criteria were included in the retrospective analysis: age over 18 years, Chinese ethnic origin, life expectancy over 6 months, histological diagnosis of invasive breast cancer by core needle biopsy or surgery, requirement for chemotherapy according to the National Comprehensive Cancer Network (NCCN), an ECOG performance status of 0-1, and the absence of fever for more than 24 hrs before the start of chemotherapy. Pregnant women, patients with second primary malignant carcinomas or who had previously received chemotherapy or radiotherapy were excluded.

### 2.3. Treatment and Follow-Up

The enrolled patients were treated with chemotherapy according to NCCN. Before or during chemotherapy cycles, the white blood cells and absolute neutrophil count of these were recorded in succession. When diagnosed with chemotherapy-induced myelosuppression (leukopenia or neutropenia), patients in Guangdong Hospital of Traditional Chinese Medicine (group 1), received TCM decoctions for jian pi (to regulate the gastrointestinal function for better assimilation) and yi qi yang xue sheng sui (to enhance the hematopoietic activity of the bone marrow), while the patients in Sun Yat-sen Memorial Hospital (group 2) did not.

When severe myelosuppression occurred (i.e., leukocyte lower than 2.0 × 10^9^/L or neutrophils lower than 1.0 × 10^9^/L), group 1 patients received the treatment of granulocyte colony-stimulating factor (G-CSF), in addition to TCM decoctions. In group 2, these patients received only G-CSF treatment. Meanwhile, body temperatures of breast cancer patients should be recorded. FN (i.e., body temperature ≥38.2°C and absolute neutrophil count <0.5 × 10^9^/L on the same day of the fever or the day after) was defined according to the definition used by the Infectious Disease Society of America (IDSA) and the European Organization for Research and Treatment of Cancer (EORTC) [10, 11].

The data of alanine transaminase (ALT), aspartate aminotransferase (AST), blood urea nitrogen (BUN), and creatinine (CR) were recorded in every chemotherapy cycle to evaluate the toxicity of TCM.

### 2.4. Statistical Analysis

All demographic and clinicopathological data had been prospectively collected in computer databases before this retrospective analysis. Continuous variables were expressed as the mean. Categorical variables were reported as the number and percentage. Differences between continuous data were analyzed using Mann-Whitney test. Differences between categorical data were analyzed using the *χ* ^2^ test. Significant risk factors associated with the occurrence of chemotherapy-induced leukopenia, neutropenia, and FN were identified using logistic regression model multivariate analysis. Correlation power of all the risk factors was evaluated by the odds ratio (OR). SPSS 17.0 statistical package (SPSS, Inc., Chicago, IL, USA) was used for the statistical analyses.

To reduce bias in patient selection, propensity analysis was carried out using logistic regression to create propensity scores for the treatment and control patients. Logistic regression was applied to the studied clinical variables differing significantly between the treatment and control patients with chemotherapy-induced myelosuppression and FN, and propensity scores were generated along a continuous range from 0 to 1. The model was then used to provide a one-to-one nearest-neighbor match between patients undergoing treatment or control. The propensity analysis was established using the matching package within R 3.0.2 software which was used for the statistical analyses [12].

All reported *P* values were those of two-sided tests. The statistical significance was set at *P* < 0.05.

## 3. Results

### 3.1. Clinical and Demographic Characteristics of the Breast Cancer Patients

**Figure 1:**
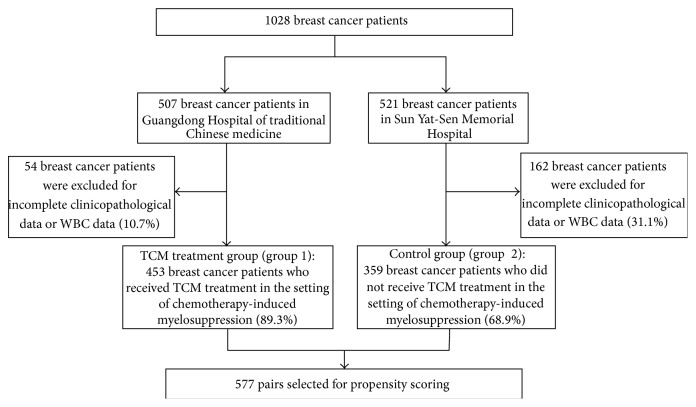
Flow diagram of the study. The patients databases included 1028 breast cancer patients. In TCM treatment group (group 1), 453 breast cancer patients received TCM treatment in the setting of chemotherapy-induced myelosuppression. In control group (group 2), 359 breast cancer patients did not receive TCM treatment in the setting of chemotherapy-induced myelosuppression. In the propensity score model, 577 pairs of matched patients were generated for baseline-adjusted analyses.

On the basis of the inclusion criteria, 812 breast cancer patients were enrolled in this retrospective study. Of these patients, 453 received TCM treatment and 359 underwent no TCM treatment (Figure 1). Baseline demographic and clinicopathological data were summarized in Table 1. There were significant differences in age (*P* = 0.000004), chemotherapy regimens (*P* < 0.0001), receiving preoperative chemotherapy (*P* = 0.0029), status of estrogen receptor (ER) (*P* = 0.000149), progesterone receptor (PR) (*P* = 0.000244), human epidermal growth factor receptor-2 (HER-2) (*P* = 0.003203), and Ki67 (*P* < 0.0001) between the two groups. There was no significant difference in tumor stage (*P* = 0.4594). Propensity analysis based on variables associated with the therapeutic strategy and myelosuppression identified 577 matched pairs of patients from each group. When only these pairs were considered, the two groups did not exhibit significant baseline difference in age, chemotherapy regimens, tumor stage, receiving preoperative chemotherapy, and status of ER, PR, HER-2, Ki67, and AST (Table 1).

### 3.2. TCM Decreased the Rate of Chemotherapy-Induced Leukopenia, Neutropenia, and FN in the Entire Study Population

**Figure 2:**
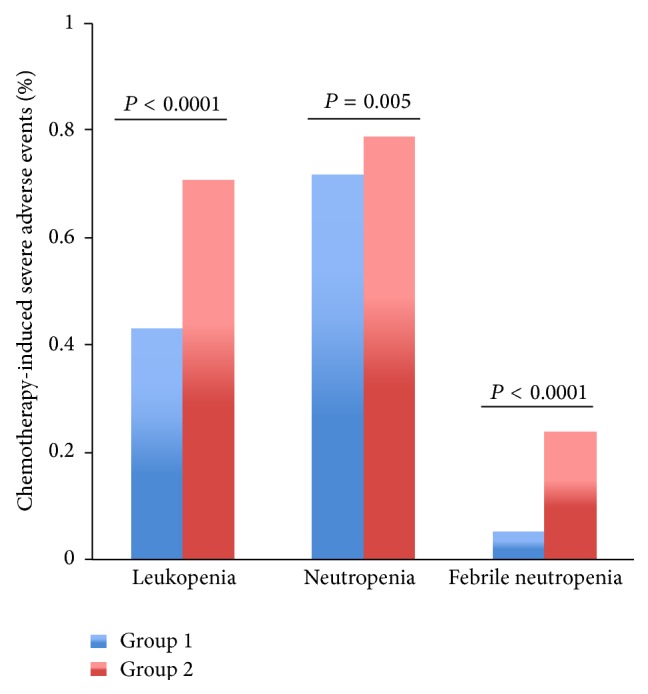
The rates of severe chemotherapy-induced leukopenia, neutropenia (Grades III-IV), and FN for the entire propensity-matched patients. After matching, patients in group 2 had a significantly higher rate of severe leukopenia, neutropenia, and FN compared to patients in group 1 (43% versus 71%, *P* < 0.0001; 72% versus 79%, *P* = 0.005; 6% versus 24%, *P* < 0.0001, resp.).

When receiving chemotherapy, there were significant differences between the two groups with respect to chemotherapy-induced leukopenia, neutropenia, and FN (Table 2). Patients in group 1 had a significantly lower rate of severe leukopenia and neutropenia compared with the patients in group 2 (43% versus 71%, *P* < 0.0001; 72% versus 79%, *P* = 0.005, resp.) (Figure 2). Furthermore, patients selected in the propensity-matching model who underwent TCM treatment exhibited a significantly lower rate of FN compared with those who did not undergo TCM treatment (6% versus 24%, *P* < 0.0001) (Figure 2).

### 3.3. Multivariate Analysis of Clinicopathological Factors Predictive of Chemotherapy-Induced Leukopenia, Neutropenia, and FN

Significant risk factors associated with the occurrence of chemotherapy-induced leukopenia, neutropenia, and FN were identified using logistic regression analysis. The variables in the multivariate model included age, chemotherapy chemotherapy regimens, tumor stage, pathologic features, and other factors (Tables 3–5).

The multivariate analysis revealed that chemotherapy regimens containing anthracyclines combined with paclitaxel or docetaxel associated with a significant risk of chemotherapy-induced leukopenia (OR 3.208, 95% CI 2.228–4.617, *P* < 0.0001), neutropenia (OR 4.184, 95% CI 2.725–6.424, *P* < 0.0001), and FN (OR 4.304, 95% CI 2.641–7.015, *P* < 0.0001) in the total population. The tumor stage associated with a significant risk of leukopenia (stage II, OR 1.360, 95% CI 1.014–1.823, *P* = 0.04; stages III and IV, OR 1.953, 95% CI 1.215–3.141, *P* = 0.006) and neutropenia (stage II, OR 1.554, 95% CI 1.139–2.120, *P* = 0.005) but not the risk of FN. The Ki67 index could increase the risk of leukopenia (OR 1.552, 95% CI 1.163–2.071, *P* = 0.003) but not the risk of neutropenia and FN. PR could increase the risk of FN (OR 2.631, 95% CI 1.418–4.882, *P* = 0.002) but not the risk of leukopenia and neutropenia.

Factor protected against chemotherapy-induced leukopenia, neutropenia, and FN was TCM treatment. In the propensity-matching model, the OR of leukopenia, neutropenia, and FN for the TCM treatment were 0.285 (95% CI 0.218–0.373, *P* < 0.0001), 0.741 (95% CI 0.554–0.992, *P* = 0.044), and 0.184 (95% CI, 0.122–0.279, *P* < 0.0001), respectively.

The factors, such as younger age and not receiving preoperative chemotherapy, could decrease the risk of leukopenia but not the risk of neutropenia and FN. The OR were 0.979 (95% CI, 0.965–0.994, *P* = 0.005) and 0.584 (95% CI, 0.377–0.905, *P* = 0.016), respectively.

No other characteristics were associated with increased risk of leukopenia, neutropenia, and FN: ER and HER-2 (*P* > 0.05).

### 3.4. Subgroup Analysis of the Entire Study Population

To explore more deeply the efficacy of chemotherapy regimens and tumor stage for chemotherapy-induced leukopenia, neutropenia, and FN, we performed subgroup analysis.

### 3.5. Subgroup Analysis by Chemotherapy Regimens

Patients in each group were divided into subgroups with different chemotherapy regimens. TCM treatment showed a benefit in relieving chemotherapy-induced leukopenia and FN in most chemotherapy regimens but little benefit in chemotherapy-induced neutropenia (see Supporting Tables 1–3 in Supplementary Material available online at http://dx.doi.org/10.1155/2015/736197).

**Figure 3:**
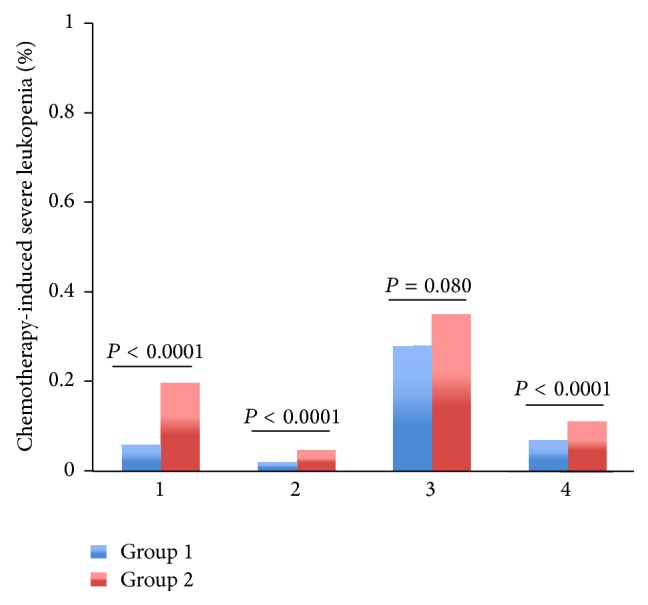
The rates of severe chemotherapy-induced leukopenia (Grades III-IV) for the entire propensity-matched patients in different chemotherapy regimens. After matching, patients in group 2 had a higher rate of severe leukopenia compared to patients in group 1 (20% versus 6%, *P* < 0.0001; 5% versus 2%, *P* < 0.0001; 35% versus 28%, *P* = 0.080; 11% versus 7%, *P* < 0.0001, resp.).* Chemotherapy*. 1: the chemotherapy regimens contain AC/EC (Adriamycin or epirubicin, Cyclophosphamide) and CAF/CEF (Adriamycin or epirubicin, Cyclophosphamide, and 5-Fluorouracil). 2: the chemotherapy regimens contain TC (paclitaxel or docetaxel, Cyclophosphamide) and T (paclitaxel or docetaxel). 3: the chemotherapy regimens contain anthracyclines combined with paclitaxel or docetaxel: TAC/TEC (paclitaxel or docetaxel, Cyclophosphamide, and Adriamycin or epirubicin); TA/TE (paclitaxel or docetaxel, Adriamycin or epirubicin). 4: the chemotherapy regimens contain anthracyclines followed by paclitaxel or docetaxel: AC/EC (Adriamycin or epirubicin, Cyclophosphamide); CAF/CEF (Adriamycin or epirubicin, Cyclophosphamide, and 5-Fluorouracil), followed with T or TH (paclitaxel or docetaxel, Herceptin).

**Figure 4:**
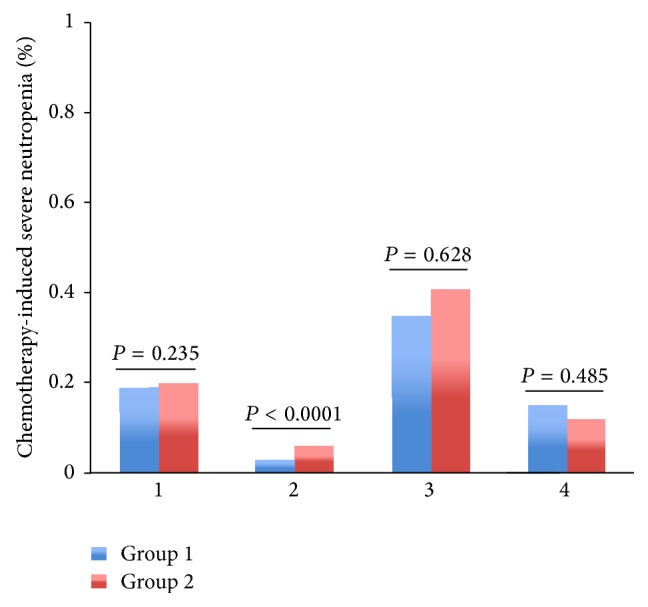
The rates of severe chemotherapy-induced neutropenia (Grades III-IV) for the entire propensity-matched patients in different chemotherapy regimens. After matching, patients in group 2 had a higher rate of severe neutropenia compared to patients in group 1 who received chemotherapy regimens 1, 2, and 3 treatment (20% versus 19%, *P* = 0.235; 6% versus 3%, *P* < 0.0001; 41% versus 35%, *P* = 0.628, resp.). However, patients in group 1 had a higher rate of severe neutropenia compared to patients in group 2 who received chemotherapy regimen 4 treatment (15% versus 12%, *P* = 0.485).* Chemotherapy*. 1: the chemotherapy regimens contain AC/EC (Adriamycin or epirubicin, Cyclophosphamide), CAF/CEF (Adriamycin or epirubicin, Cyclophosphamide, and 5-Fluorouracil). 2: the chemotherapy regimens contain TC (paclitaxel or docetaxel, Cyclophosphamide) and T (paclitaxel or docetaxel). 3: the chemotherapy regimens contain anthracyclines combined with paclitaxel or docetaxel: TAC/TEC (paclitaxel or docetaxel, Cyclophosphamide, and Adriamycin or epirubicin); TA/TE (paclitaxel or docetaxel, Adriamycin or epirubicin). 4: the chemotherapy regimens contain anthracyclines followed by paclitaxel or docetaxel: AC/EC (Adriamycin or epirubicin, Cyclophosphamide); CAF/CEF (Adriamycin or epirubicin, Cyclophosphamide, and 5-Fluorouracil), followed with T or TH (paclitaxel or docetaxel, Herceptin).

**Figure 5:**
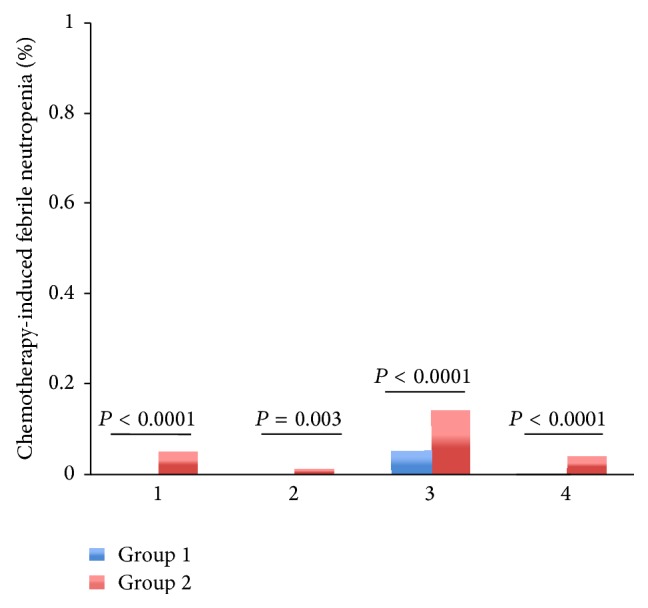
The rates of chemotherapy-induced FN for the entire propensity-matched patients in different chemotherapy regimens. After matching, patients in group 2 had a significantly higher rate of FN compared to patients in group 1 (5% versus 0%, *P* < 0.0001; 1% versus 0%, *P* = 0.003; 14% versus 5%, *P* < 0.0001; 4% versus 0%, *P* < 0.0001, resp.).* Chemotherapy*: 1: the chemotherapy regimens contain AC/EC (Adriamycin or epirubicin, Cyclophosphamide) and CAF/CEF (Adriamycin or epirubicin, Cyclophosphamide, and 5-Fluorouracil). 2: the chemotherapy regimens contain TC (paclitaxel or docetaxel, Cyclophosphamide) and T (paclitaxel or docetaxel). 3: the chemotherapy regimens contain anthracyclines combined with paclitaxel or docetaxel: TAC/TEC (paclitaxel or docetaxel, Cyclophosphamide, and Adriamycin or epirubicin); TA/TE (paclitaxel or docetaxel, Adriamycin or epirubicin). 4: the chemotherapy regimens contain anthracyclines followed by paclitaxel or docetaxel: AC/EC (Adriamycin or epirubicin, Cyclophosphamide); CAF/CEF (Adriamycin or epirubicin, Cyclophosphamide, and 5-Fluorouracil), followed with T or TH (paclitaxel or docetaxel, Herceptin).

Among patients with regimens containing paclitaxel or docetaxel, TCM provided lower rate of severe leukopenia (2% versus 5%, *P* = 0.000006), neutropenia (3% versus 6%, *P* = 0.000012), and FN (0% versus 1%, *P* = 0.003053) than control group (Figures 3–5). Among patients with regimens containing anthracyclines only and regimens containing anthracyclines followed by paclitaxel or docetaxel, TCM provided lower severe leukopenia (6% versus 20%, *P* < 0.0001; 7% versus 11%, *P* = 0.000034, resp.) and FN (0% versus 5%, *P* < 0.0001; 0% versus 4%, *P* < 0.0001, resp.), but there was no significant difference in severe neutropenia (19% versus 20%, *P* = 0.235; 15% versus 12%, *P* = 0.485, resp.) (Figures 3–5). Among patients with regimens containing anthracyclines combined with paclitaxel or docetaxel, TCM provided lower rate of FN (5% versus 14%, *P* = 0.000001), but there was no significant difference in severe leukopenia (28% versus 35%, *P* = 0.08) and neutropenia (35% versus 41%, *P* = 0.628) (Figures 3–5).

### 3.6. Subgroup Analysis by Tumor Stage

**Figure 6:**
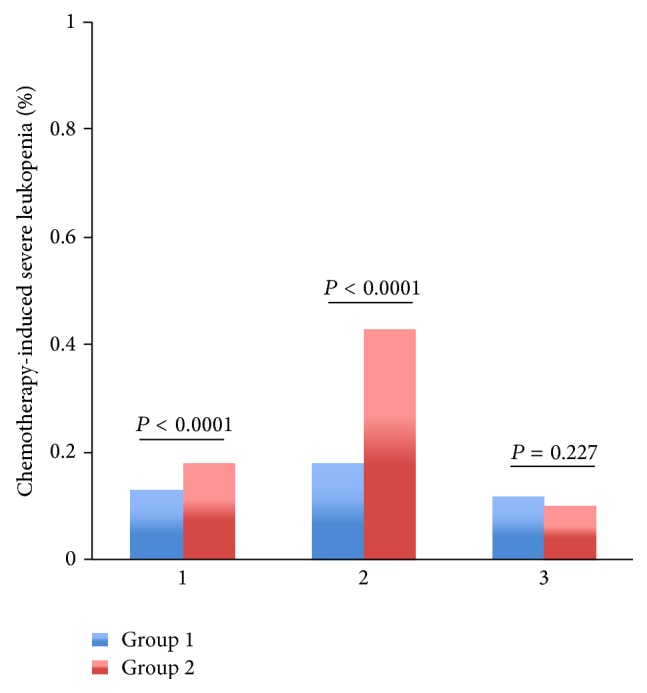
The rates of severe chemotherapy-induced leukopenia (Grades III-IV) for the entire propensity-matched patients in different stages. In stages 1 and 2, patients in group 2 had a significantly higher rate of severe leukopenia compared to patients in group 1 (18% versus 13%, *P* < 0.0001; 43% versus 18%, *P* < 0.0001, resp.). However, in stage 3, patients in group 1 had a higher rate of severe leukopenia compared to patients in group 2 (12% versus 10%, *P* = 0.227). Tumor stage: 1 indicates stage I; 2 indicates stage II; 3 indicates stages III and IV.

**Figure 7:**
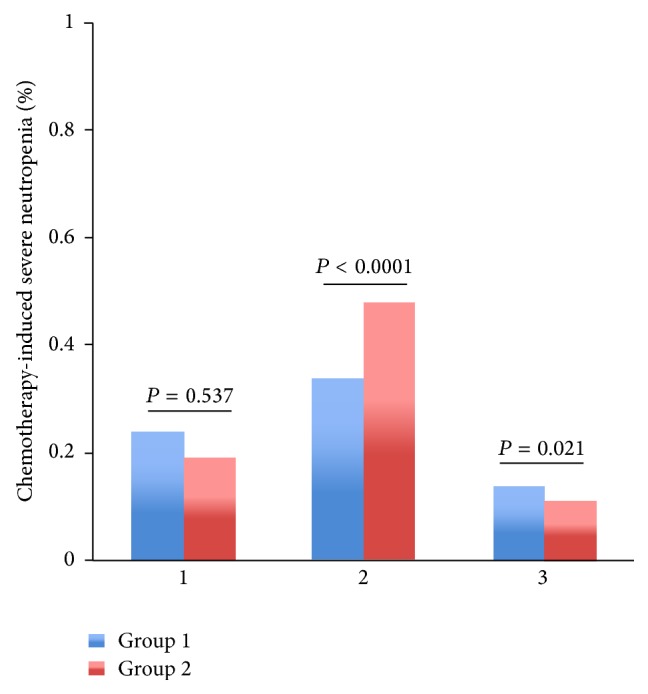
The rates of severe chemotherapy-induced neutropenia (Grades III-IV) for the entire propensity-matched patients in different stages. After matching, in stages 1 and 3, patients in group 1 had a higher rate of severe neutropenia compared to patients in group 2 (24% versus 19%, *P* = 0.537; 14% versus 11%, *P* = 0.021, resp.). However, in stage 2, patients in group 2 had a significantly higher rate of severe neutropenia compared to patients in group 1 (48% versus 34%, *P* < 0.0001). Tumor stage: 1 indicates stage I; 2 indicates stage II; 3 indicates stages III and IV.

**Figure 8:**
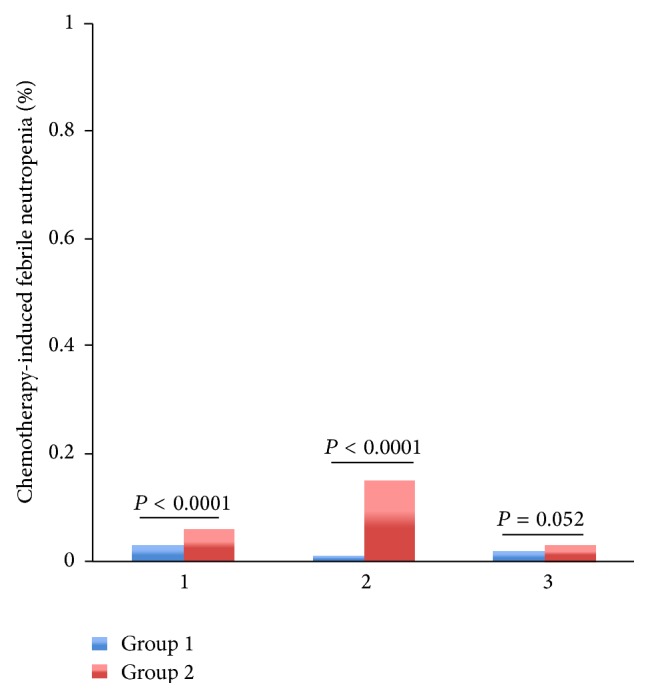
The rates of chemotherapy-induced FN for the entire propensity-matched patients in different stages. After matching, patients in group 2 had a higher rate of FN compared to patients in group 1 (6% versus 3%, *P* < 0.0001; 15% versus 1%, *P* < 0.0001; 3% versus 2%, *P* = 0.052, resp.). Tumor stage: 1 indicates stage I; 2 indicates stage II; 3 indicates stages III and IV.

Patients were divided into subgroups with tumor stage. Among patients at stage II, severe leukopenia (18% versus 43%, *P* < 0.0001), severe neutropenia (34% versus 48%, *P* = 0.000093), and FN (1% versus 15%, *P* < 0.0001) were significantly lower in group 1 (Supporting Tables 4–6) (Figures 6–8). Among patients at stage I, severe leukopenia (13% versus 18%, *P* < 0.0001) and FN (3% versus 6%, *P* = 0.000152) were significantly lower in group 1. But there was no significant difference in severe neutropenia (24% versus 19%, *P* = 0.537) between two groups (Supporting Tables 4–6) (Figures 6–8). Among patients at stage III, TCM treatment showed little effect in relieving chemotherapy-induced severe leukopenia (12% versus 10%, *P* = 0.227), severe neutropenia (14% versus 11%, *P* = 0.021), and FN (2% versus 3%, *P* = 0.052) (Supporting Tables 4–6) (Figures 6–8).

### 3.7. The Toxicity of TCM

There was no patient suffering from renal damage during the chemotherapy in TCM treatment group. Hepatic dysfunction could be found in both groups. The numbers of patients in group 1 and group 2 concomitant with ALT elevation were 71 and 102, respectively. However, the numbers of patients in group 1 and group 2 concomitant with AST elevation were 59 and 78, respectively.

After propensity matching, the rates of ALT elevation were 16% in group 1 and 23% in group 2 (*P* = 0.004167). The rates of AST elevation were 14% in group 1 and 16% in group 2 (*P* = 0.0224) (Supporting Table 7).

## 4. Discussion

Chemotherapy-induced myelosuppression is an important and often dose-limiting toxicity of cytotoxic anticancer agents. It may lower the quality of life in breast cancer patients and be associated with many complications, including increased risks for opportunistic infections, FN, sepsis, and mortality.

TCM is considered as an appropriate therapy for managing chronic diseases such as cancer but mainly for symptom relief or palliative care rather than for curing the disease. Previous studies had shown that some TCMs could reduce the side effects of chemotherapy, modulate immune effector cells, and relieve chemotherapy-induced myelosuppression [13–16]. Researchers explained the effectiveness of some herbs on neutrophils by biochemical tests and then attributed these to [17–20] (i) improving the bone marrow hematopoietic microenvironment; (ii) improving the cyclin D1 expression, promoting cell cycles in the G0/G1 phase to enter the S, G2/M phases to accelerate hematopoietic progenitor cell proliferation and differentiation, and involving the PI3K/AKT pathway to thereby prevent bone marrow nucleated cells from apoptosis; and (iii) regulating the immune function and the expression of CDK4 and CDK6 in bone marrow and tumor tissues, stimulating the expression of IL-1*β*, IL-3, IL-6, SCF, and GM-CSF and inhibiting the expression of TGF-*β*.

The clinical studies on the effects of TCM on chemotherapy-induced myelosuppression in breast cancer patients were little. Consequently, we proposed this research by treating myelosuppression patients with TCM and G-CSF compared with G-CSF alone. We demonstrated that TCM treatment provided an absolute risk reduction of severe leukopenia events by 28% (*P* < 0.0001). Furthermore, TCM treatment also reduced the risk of severe neutropenia events by 7% (*P* = 0.005) and reduced the risk of FN event by 18% (*P* < 0.0001) in breast cancer patients. Previous studies had shown that the morbidity of FN in treatment-naive patients was approximately 15–40% [21, 22]. While, in our study, 24% of the patients in the control group developed FN, however, in the TCM treatment group only 6% of the patients did. This result might indicate that TCM was another good choice for chemotherapy-induced myelosuppression patients, especially for patients who suffered from G-CSF intolerance or allergy. However, the effects of TCM on leukopenia, neutropenia, and FN were not exactly the same. In our study, TCM treatment showed more benefits in relieving chemotherapy-induced leukopenia and FN in most patients but just acted on chemotherapy-induced neutropenia in some patients. Among patients with regimens containing anthracyclines combined with paclitaxel or docetaxel, TCM gave no relief from leukopenia and neutropenia but provided lower rate of FN compared with control group. These data suggested that the effects of TCM on myelosuppression may not be fully in accord with G-CSF but may be achieved by increasing neutrophilic granulocytes and other immunocytes.

In previous studies, older age, lower weight, and a higher planned dose intensity of doxorubicin, epirubicin, or docetaxel were risk factors for severe neutropenia or FN [23–25]. PR status, HER-2 status, lymphovascular invasion, comorbidities, smoking status, alcohol usage, hemoglobin levels, platelet/absolute lymphocyte/absolute monocyte counts, and creatinine level had little effect. These findings were incompletely consistent with our study.

In our trial, the multivariate analysis demonstrated that chemotherapy regimens containing anthracyclines combined with paclitaxel or docetaxel received by patients were the most significant predictor for chemotherapy-induced leukopenia, neutropenia, and FN. Tumor stage and receiving preoperative chemotherapy might be associated with the risk of leukopenia. A possible explanation was that patients in later stages were more likely treated with preoperative chemotherapy and a dose intensity regimen of doxorubicin, epirubicin, or docetaxel. These patients should be treated with G-CSF or TCM prophylaxis.

Although some previous studies had shown that factors such as age [21, 22] and ER positivity were associated with the risk of FN [25], our results did not support those findings. This might be because the patients in our study were very young (50 years old) and in good conditions. In our trial, the ER, PR, and HER-2 status had little effect on the risks of chemotherapy-induced myelosuppression and FN, whereas Ki-67 positivity increased the risk of leucocytes. It was possible that Ki-67 positivity correlated with the degree of malignancy and that patients would be more likely to be treated with preoperative chemotherapy and a dose intensity regimen of doxorubicin, epirubicin, or docetaxel. As to HER-2, one possible explanation was that patients with HER-2 positivity were more likely to be treated with chemotherapy regimen which contains anthracyclines followed by paclitaxel or docetaxel and Herceptin, if not the result of chance.

Multivariate analyses had confirmed the effect of TCM treatment on chemotherapy-induced myelosuppression and FN. For most Chinese patients, TCM was much more easily accessed and maintained than G-CSF. In addition, TCM was convenient to use because of its oral administration and lack of adverse events, as in our study it showed little hepatic or renal dysfunction, and it was cheap. In fact, TCM could be used alone or in combination with pharmacological agents, which might improve the efficacy and decrease adverse events. TCM treatment might be an optimal alternative therapy for chemotherapy-induced myelosuppression and FN patients. But our data also indicated that TCM showed little effect on patients at stage III and received regimens containing anthracyclines combined with paclitaxel or docetaxel. In our view, these patients were in serious condition, often bone marrow micrometastasis, which might damage hematopoietic function. In addition, the chemotherapy would cause severe nausea and vomiting, poor appetite, dyssomnia, and suppressed assimilation, which lead to hemopoiesis injured ultimately.

Because our study is a retrospective analysis, a randomized, controlled study is necessary to confirm our results. And some molecular biology experiments are needed to identify the molecular action of TCM on myelosuppression and FN.

## Supplementary Material

This supplementary material was to show the efficacy of chemotherapy regimens and tumor staging for chemotherapy-induced leukopenia, neutropenia and FN more deeply.
In Supporting Table 1–3, Patients in TCM treatment showed a benefit in relieving chemotherapy-induced leukopenia and FN in most chemotherapy regimens in various degrees, but little benefit in chemotherapy-induced neutropenia.
In Supporting Table 4–6, among patients at stage I or II, severe leukopenia, and FN were significantly lower in TCM treatment group. Among patients at stage III, TCM treatment showed little effect.
As to the toxicity of TCM, there was no patient suffering from renal damage during the chemotherapy in TCM treatment group. Hepatic dysfunction could be found in both groups, and there was no significant difference.

## Figures and Tables

**Table 1:** Clinicopathological data of breast cancer patients.

Variables	Before propensity matching			After propensity matching		
	Group 1 (*n* = 453)	Group 2 (*n* = 359)	*P*	Group 1 (*n* = 577)	Group 2 (*n* = 577)	*P*
Age, yr	50.3 (49.4–51.2)	47.1 (46.1–48.1)	**0.000004**	49.8 (49.0–50.6)	50.2 (49.3–51.0)	0.504
Chemotherapy			***<*0.0001**			0.250
1	154	35		179	173	
2	42	54		63	52	
3	172	183		229	260	
4	85	87		106	92	
TNM stage			0.4594			0.200
I	164	117		215	164	
II	219	189		275	333	
III and IV	70	53		87	80	
Neoadjuvant chemotherapy			0.0029			0.637
Yes	73	88		94	100	
No	380	271		483	477	
ER			**0.000149**			0.899
−	146	73		179	177	
+	307	286		398	400	
PR			**0.000244**			0.811
−	193	108		237	241	
+	260	251		340	336	
HER-2			**0.003203**			0.052
−	340	300		441	468	
+	113	59		136	109	
Ki67			***<*0.0001**			0.813
−	256	93		304	308	
+	197	266		273	269	
ALT (U/L)			**0.000038**			**0.008**
≤40	382	257		487	446	
>40	71	102		90	131	
AST (U/L)			**0.004**			0.224
≤40	394	281		499	483	
>40	59	78		78	94	

HER-2 positive: testing by immunohistochemical (IHC) assay (3+) or in situ hybridization (ISH) assay (+); HER-2 negative: IHC (−), (1+), and (2+) or ISH (−); ALT: alanine aminotransferase; AST: aspartate transferase. Chemotherapy: 1: the chemotherapy regimens contain AC/EC (Adriamycin or epirubicin, Cyclophosphamide) and CAF/CEF (Adriamycin or epirubicin, Cyclophosphamide, and 5-Fluorouracil). 2: the chemotherapy regimens contain TC (paclitaxel or docetaxel, Cyclophosphamide) and T (paclitaxel or docetaxel). 3: the chemotherapy regimens contain anthracyclines combined with paclitaxel or docetaxel: TAC/TEC (paclitaxel or docetaxel, Cyclophosphamide, and Adriamycin or epirubicin); TA/TE (paclitaxel or docetaxel, Adriamycin or epirubicin). 4: the chemotherapy regimens contain anthracyclines followed by paclitaxel or docetaxel: AC/EC (Adriamycin or epirubicin, Cyclophosphamide); CAF/CEF (Adriamycin or epirubicin, Cyclophosphamide, and 5-Fluorouracil), followed with T or TH (paclitaxel or docetaxel, Herceptin).

**Table 2:** Different outcomes in chemotherapy-induced leukopenia, neutropenia, and FN between two groups.

Outcomes	Before propensity matching			After propensity matching		
	Group 1 (*n* = 453)	Group 2 (*n* = 359)	*P*	Group 1 (*n* = 577)	Group 2 (*n* = 577)	*P*
Leukopenia			<**0.0001**			<**0.0001**
Mild	261	85		328	169	
Severe	192	274		249	408	
Neutropenia			**0.000842**			**0.005**
Mild	126	64		163	122	
Severe	327	295		414	455	
Febrile neutropenia			<**0.0001**			<**0.0001**
No	428	254		545	439	
Yes	25	105		32	138	

Mild: Grades I and II myelosuppression. Leukocyte lower than normal but higher than (or equal to) 2.0 × 10^9^/L; neutrophils lower than normal but higher than (or equal to) 1.0 × 10^9^/L. Severe: Grades III and IV myelosuppression. Leukocyte lower than 2.0 × 10^9^/L; neutrophils lower than 1.0 × 10^9^/L. FN: body temperature ≥38.2°C and absolute neutrophil count <0.5 × 10^9^/L on the same day of the fever or the day after.

**Table 3:** Multivariate analysis of clinicopathological variables of leukopenia.

Variables	Before propensity matching			After propensity matching		
	OR	95% CI	*P*	OR	95% CI	*P*
Age	0.989	0.971–1.006	0.199	0.979	0.965–0.994	**0.005**
Chemotherapy regimens						
1	1	—	—	1	—	—
2	0.976	0.539–1.765	0.935	0.773	0.468–1.274	0.312
3	4.477	2.764–7.251	***<*0.0001**	3.208	2.228–4.617	***<*0.0001**
4	1.552	0.942–2.558	0.085	1.251	0.837–1.869	0.275
TNM stage						
I	1	—	—	1	—	—
II	1.227	0.858–1.756	0.262	1.360	1.014–1.823	**0.040**
III and IV	2.436	1.362–4.357	**0.003**	1.953	1.215–3.141	**0.006**
Neoadjuvant chemotherapy	0.788	0.476–1.304	0.354	0.584	0.377–0.905	**0.016**
ER	1.165	0.655–2.071	0.603	1.215	0.772–1.913	0.399
PR	0.781	0.457–1.333	0.364	1.003	0.654–1.537	0.989
HER-2	0.857	0.561–1.307	0.473	1.094	0.762–1.572	0.626
Ki67	1.132	0.791–1.620	0.499	1.552	1.163–2.071	**0.003**
TCM treatment	0.252	0.172–0.367	***<*0.0001**	0.285	0.218–0.373	***<*0.0001**

Chemotherapy: 1: the chemotherapy regimens contain AC/EC (Adriamycin or epirubicin, Cyclophosphamide) and CAF/CEF (Adriamycin or epirubicin, Cyclophosphamide, and 5-Fluorouracil). 2: the chemotherapy regimens contain TC (paclitaxel or docetaxel, Cyclophosphamide) and T (paclitaxel or docetaxel). 3: the chemotherapy regimens contain anthracyclines combined with paclitaxel or docetaxel: TAC/TEC (paclitaxel or docetaxel, Cyclophosphamide, and Adriamycin or epirubicin); TA/TE (paclitaxel or docetaxel, Adriamycin or epirubicin). 4: the chemotherapy regimens contain anthracyclines followed by paclitaxel or docetaxel: AC/EC (Adriamycin or epirubicin, Cyclophosphamide); CAF/CEF (Adriamycin or epirubicin, Cyclophosphamide, and 5-Fluorouracil), followed with T or TH (paclitaxel or docetaxel, Herceptin).

**Table 4:** Multivariate analysis of clinicopathological variables of neutropenia.

Variables	Before propensity matching			After propensity matching		
	OR	95% CI	*P*	OR	95% CI	*P*
Age	1.006	0.987–1.026	0.516	0.998	0.982–1.014	0.777
Chemotherapy regimens						
1	1	—	—	1	—	—
2	0.592	0.336–1.045	0.071	0.580	0.361–0.931	**0.024**
3	3.853	2.245–6.614	**0.000001**	4.184	2.725–6.424	***<*0.0001**
4	2.124	1.252–3.606	**0.005**	1.984	1.282–3.072	**0.002**
TNM stage						
I	1	—	—	1	—	—
II	1.157	0.797–1.678	0.443	1.554	1.139–2.120	**0.005**
III and IV	2.029	0.989–4.164	0.054	1.683	0.952–2.967	**0.073**
Neoadjuvant chemotherapy	0.962	0.523–1.768	0.901	0.896	0.521–1.541	0.691
ER	1.134	0.600–2.144	0.698	0.932	0.556–1.561	0.789
PR	0.957	0.533–1.718	0.883	0.969	0.602–1.560	0.898
HER-2	0.962	0.599–1.546	0.873	1.090	0.722–1.645	0.682
Ki67	0.859	0.582–1.266	0.442	1.022	0.745–1.402	0.893
TCM treatment	0.607	0.400–0.923	**0.019**	0.741	0.554–0.992	**0.044**

Chemotherapy: 1: the chemotherapy regimens contain AC/EC (Adriamycin or epirubicin, Cyclophosphamide) and CAF/CEF (Adriamycin or epirubicin, Cyclophosphamide, and 5-Fluorouracil). 2: the chemotherapy regimens contain TC (paclitaxel or docetaxel, Cyclophosphamide) and T (paclitaxel or docetaxel). 3: the chemotherapy regimens contain anthracyclines combined with paclitaxel or docetaxel: TAC/TEC (paclitaxel or docetaxel, Cyclophosphamide, and Adriamycin or epirubicin); TA/TE (paclitaxel or docetaxel, Adriamycin or epirubicin). 4: the chemotherapy regimens contain anthracyclines followed by paclitaxel or docetaxel: AC/EC (Adriamycin or epirubicin, Cyclophosphamide); CAF/CEF (Adriamycin or epirubicin, Cyclophosphamide, and 5-Fluorouracil), followed with T or TH (paclitaxel or docetaxel, Herceptin).

**Table 5:** Multivariate analysis of clinicopathological variables of FN.

Variables	Before propensity matching			After propensity matching		
	OR	95% CI	*P*	OR	95% CI	*P*
Age	0.985	0.963–1.007	0.182	0.995	0.979–1.014	**0.590**
Chemotherapy regimens						
1	1	—	—	1	—	—
2	1.797	0.585–5.514	0.306	0.785	0.321–1.918	**0.595**
3	6.433	2.612–15.844	**0.000052**	4.304	2.641–7.015	***<*0.0001**
4	2.516	0.942–6.721	0.066	1.917	1.035–3.552	**0.039**
TNM stage						
I	1	—	—	1	—	—
II	0.895	0.542–1.478	0.665	0.925	0.613–1.396	**0.711**
III and IV	0.897	0.457–1.757	0.750	0.690	0.383–1.244	**0.217**
Neoadjuvant chemotherapy	0.710	0.426–1.184	**0.190**	1.519	0.916–2.522	**0.106**
ER	0.588	0.289–1.195	**0.142**	0.544	0.291–1.014	0.055
PR	1.404	0.720–2.738	0.319	2.631	1.418–4.882	0.002
HER-2	0.803	0.464–1.391	0.434	1.009	0.622–1.637	**0.970**
Ki67	0.946	0.589–1.520	0.819	1.182	0.812–1.721	0.383
TCM treatment	0.166	0.098–0.280	***<*0.0001**	0.184	0.122–0.279	***<*0.0001**

Chemotherapy: 1: the chemotherapy regimens contain AC/EC (Adriamycin or epirubicin, Cyclophosphamide) and CAF/CEF (Adriamycin or epirubicin, Cyclophosphamide, and 5-Fluorouracil). 2: the chemotherapy regimens contain TC (paclitaxel or docetaxel, Cyclophosphamide) and T (paclitaxel or docetaxel). 3: the chemotherapy regimens contain anthracyclines combined with paclitaxel or docetaxel: TAC/TEC (paclitaxel or docetaxel, Cyclophosphamide, and Adriamycin or epirubicin); TA/TE (paclitaxel or docetaxel, Adriamycin or epirubicin). 4: the chemotherapy regimens contain anthracyclines followed by paclitaxel or docetaxel: AC/EC (Adriamycin or epirubicin, Cyclophosphamide); CAF/CEF (Adriamycin or epirubicin, Cyclophosphamide, and 5-Fluorouracil), followed with T or TH (paclitaxel or docetaxel, Herceptin). FN: body temperature ≥38.2°C and absolute neutrophil count <0.5 × 10^9^/L on the same day of the fever or the day after.
