# Supplementary material for: Effects of Traditional Chinese Medicine on Chemotherapy-Induced Myelosuppression and Febrile Neutropenia in Breast Cancer Patients
Source: Evid Based Complement Alternat Med. 2015 Aug 12;2015:736197. doi: 10.1155/2015/736197 (PMC4549530; doi:10.1155/2015/736197)
Supplement: Supplementary file 1 — This supplementary material was to show the efficacy of chemotherapy regimens and tumor staging for chemotherapy-induced leukopenia, neutropenia and FN more deeply. In Supporting Table 1–3, Patients in TCM treatment showed a benefit in relieving chemotherapy-induced leukopenia and FN in most chemotherapy regimens in various degrees, but little benefit in chemotherapy-induced neutropenia. In Supporting Table 4–6, among patients at stage I or II, severe leukopenia, and FN were significantly lower in TCM treatment group. Among patients at stage III, TCM treatment showed little effect. As to the toxicity of TCM, there was no patient suffering from renal damage during the chemotherapy in TCM treatment group. Hepatic dysfunction could be found in both groups, and there was no significant difference. [file 736197.f1.pdf]

Supporting Table 1 Subgroups of Chemotherapy Regiments with Different Outcomes In Chemotherapy-Induced Leukopenia Between Two Groups

Outcomes	Before Propensity Matching			After Propensity Matching		
	Group1（n）	Group2（n）	*P*	Group1（n）	Group2（n）	*P*
**Chemotherapy regiments 1**						
**Mild**	121	10	**<0.0001**	142	58	**<0.0001**
**Severe**	33	25		37	115	
**Chemotherapy regiments 2**						
**Mild**	35	22	**0.000025**	54	24	**0.000006**
**Severe**	7	32		9	28	
**Chemotherapy regiments 3**						
**Mild**	52	25	**0.000153**	65	56	0.080
**Severe**	120	158		164	204	
**Chemotherapy regiments 4**						
**Mild**	53	28	**0.000074**	67	31	**0.000034**
**Severe**	32	59		39	61	

Mild: Grade Ⅰ and Ⅱ myelosuppression. **Severe:** Grade Ⅲ and Ⅳ myelosuppression. **Chemotherapy:**

1 The chemotherapy contains AC/EC (Adriamycin or Epirubicin, Cyclophosphamide), CAF/CEF(Adriamycin or Epirubicin, Cyclophosphamide, 5-Fluorouracil).

2 The chemotherapy contains TC (Paclitaxel or Docetaxel, Cyclophosphamide), T (Paclitaxel or Docetaxel).

3 The chemotherapy contains anthracyclines combined with paclitaxel or docetaxel: TAC/TEC (Paclitaxel or Docetaxel, Cyclophosphamide, Adriamycin or Epirubicin); TA/TE (Paclitaxel or Docetaxel , Adriamycin or Epirubicin).

4 The chemotherapy contains anthracyclines followed by paclitaxel or docetaxel: AC/EC (Adriamycin or Epirubicin, Cyclophosphamide), CAF/CEF (Adriamycin or Epirubicin, Cyclophosphamide, 5-Fluorouracil), followed with T or TH(Paclitaxel or Docetaxel, herceptin).

Supporting Table 2 Subgroups of Chemotherapy Regiments with Different Outcomes In Chemotherapy-Induced Neutropenia Between Two Groups

Outcomes	Before Propensity Matching			After Propensity Matching		
	Group1 （n）	Group2 （n）	*P*	Group1 （n）	Group2 （n）	*P*
**Chemotherapy regiments 1**						
**Mild**	61	11	0.368	72	59	0.235
**Severe**	93	24		107	114	
**Chemotherapy regiments 2**						
**Mild**	27	17	**0.001**	44	15	**0.000012**
**Severe**	15	37		19	37	
**Chemotherapy regiments 3**						
**Mild**	21	18	0.475	26	26	0.628
**Severe**	151	165		203	234	
**Chemotherapy regiments 4**			0.911			
**Mild**	17	18		21	22	0.485
**Severe**	68	69		85	70	

Mild: Grade Ⅰ and Ⅱ myelosuppression. **Severe:** Grade Ⅲ and Ⅳ myelosuppression. **Chemotherapy:**

1 The chemotherapy contains AC/EC (Adriamycin or Epirubicin, Cyclophosphamide), CAF/CEF(Adriamycin or Epirubicin, Cyclophosphamide, 5-Fluorouracil).

2 The chemotherapy contains TC (Paclitaxel or Docetaxel, Cyclophosphamide), T (Paclitaxel or Docetaxel).

3 The chemotherapy contains anthracyclines combined with paclitaxel or docetaxel: TAC/TEC (Paclitaxel or Docetaxel, Cyclophosphamide, Adriamycin or Epirubicin); TA/TE (Paclitaxel or Docetaxel , Adriamycin or Epirubicin).

4 The chemotherapy contains anthracyclines followed by paclitaxel or docetaxel: AC/EC (Adriamycin or Epirubicin, Cyclophosphamide), CAF/CEF (Adriamycin or Epirubicin, Cyclophosphamide, 5-Fluorouracil), followed with T or TH(Paclitaxel or Docetaxel, herceptin).

Supporting Table 3　Subgroups of Chemotherapy Regiments with Different Outcomes In Chemotherapy-Induced FN Between Two Groups

Outcomes	Before Propensity Matching			After Propensity Matching		
	**Group1 （n）**	**Group2 （n）**	***P***	**Group1 （n）**	**Group2 （n）**	***P***
**Chemotherapy regiments 1**						
**Mild**	153	30	**0.000295**	178	144	**<0.0001**
**Severe**	1	5		1	29	
**Chemotherapy regiments 2**						
**Mild**	42	45	**0.004**	63	45	**0.003053**
**Severe**	0	9		0	7	
**Chemotherapy regiments 3**						
**Mild**	150	110	**<0.0001**	200	179	**0.000001**
**Severe**	22	73		29	81	
**Chemotherapy regiments 4**						
**Mild**	83	69	**0.000176**	104	71	**<0.0001**
**Severe**	2	18		2	21	

Mild: Grade Ⅰ and Ⅱ myelosuppression. **Severe:** Grade Ⅲ and Ⅳ myelosuppression. **Chemotherapy:**

1 The chemotherapy contains AC/EC (Adriamycin or Epirubicin, Cyclophosphamide), CAF/CEF(Adriamycin or Epirubicin, Cyclophosphamide, 5-Fluorouracil).

2 The chemotherapy contains TC (Paclitaxel or Docetaxel, Cyclophosphamide), T (Paclitaxel or Docetaxel).

3 The chemotherapy contains anthracyclines combined with paclitaxel or docetaxel: TAC/TEC (Paclitaxel or Docetaxel, Cyclophosphamide, Adriamycin or Epirubicin); TA/TE (Paclitaxel or Docetaxel , Adriamycin or Epirubicin).

4 The chemotherapy contains anthracyclines followed by paclitaxel or docetaxel: AC/EC (Adriamycin or Epirubicin, Cyclophosphamide), CAF/CEF (Adriamycin or Epirubicin, Cyclophosphamide, 5-Fluorouracil), followed with T or TH(Paclitaxel or Docetaxel, herceptin).

Supporting Table 4 Subgroups of Tumor Stage with Different Outcomes In Chemotherapy-Induced Leukopenia Between Two Groups

Outcomes	Before Propensity Matching			After Propensity Matching		
	Group1 （n）	Group2 （n）	*P*	Group1 （n）	Group2 （n）	*P*
**TNM stage Ⅰ**						
**Mild**	108	44	**0.000003**	141	63	**<0.0001**
**Severe**	56	73		74	101	
**TNM stage Ⅱ**						
**Mild**	136	32	**<0.0001**	169	83	**<0.0001**
**Severe**	83	157		106	250	
**TNM stage Ⅲ&Ⅳ**						
**Mild**	17	9	0.326	18	23	0.227
**Severe**	53	44		69	57	

Mild: Grade Ⅰ and Ⅱ myelosuppression. **Severe:** Grade Ⅲ and Ⅳ myelosuppression.

Supporting Table 5 Subgroups of Tumor Stage with Different Outcomes In Chemotherapy-Induced Neutropenia Between Two Groups

Outcomes	Before Propensity Matching			After Propensity Matching		
	Group1 （n）	Group2 （n）	*P*	Group1 （n）	Group2 （n）	*P*
**TNM stage Ⅰ**						
**Mild**	55	33	0.342	76	53	0.537
**Severe**	109	84		139	111	
**TNM stage Ⅱ**						
**Mild**	66	24	**0.000023**	81	54	**0.000093**
**Severe**	153	165		194	279	
**TNM stage Ⅲ&Ⅳ**						
**Mild**	5	7	0.262	6	15	0.021
**Severe**	65	46		81	65	

Mild: Grade Ⅰ and Ⅱ myelosuppression. **Severe:** Grade Ⅲ and Ⅳ myelosuppression.

Supporting Table 6 Subgroups of Tumor Stage with Different Outcomes In Chemotherapy-Induced FN Between Two Groups

Outcomes	Before Propensity Matching			After Propensity Matching		
	Group1 （n）	Group2 （n）	*P*	Group1 （n）	Group2 （n）	*P*
**TNM stage Ⅰ**						
**Mild**	154	90	**0.000033**	199	130	**0.000152**
**Severe**	10	27		16	34	
**TNM stage Ⅱ**						
**Mild**	212	127	**<0.0001**	268	246	**<0.0001**
**Severe**	7	62		7	87	
**TNM stage Ⅲ&Ⅳ**						
**Mild**	62	37	**0.009**	78	63	0.052
**Severe**	8	16		9	17	

Mild: Grade Ⅰ and Ⅱ myelosuppression. **Severe:** Grade Ⅲ and Ⅳ myelosuppression.

Supporting Table 7 The Toxicity of TCM

Variables	Before Propensity Matching			After Propensity Matching		
	Group1 （n=453）	Group2 （n=359）	*P*	Group1 （n=577）	Group2 （n=577）	*P*
**Renal damage**						
**BUN (mmol /L)**						
**2.14-7.14**	453	359		577	577	
**> 7.14**	0	0		0	0	
**CR (umol /L)**						
**45-84**	453	359		577	577	
**> 84**	0	0		0	0	
**Hepatic dysfunction**						
**ALT (IU/L)**						
**≤40**	382	257	**0.000842**	487	446	**0.004167**
**>40**	71	102		90	131	
**AST (IU/L)**						
**≤40**	394	281	**<0.0001**	499	483	**<0.0001**
**>40**	59	78		78	94	

ALT, alanine aminotransferase; **AST,** aspartase transferase. **BUN**, blood urea nitrogen creatinine; **CR**, creatinine.
